# Mutant Single Nucleotide Polymorphism rs189037 in Ataxia-Telangiectasia Mutated Gene Is Significantly Associated With Ventricular Wall Thickness and Human Lifespan

**DOI:** 10.3389/fcvm.2021.658908

**Published:** 2021-05-26

**Authors:** Shihui Fu, Jianqiu Hu, Xiaoping Chen, Bo Li, Hongjuan Shun, Juelin Deng, Yujie Zhang, Yao Yao, Yali Zhao

**Affiliations:** ^1^Department of Cardiology, Hainan Hospital of Chinese People's Liberation Army General Hospital, Sanya, China; ^2^Department of Geriatric Cardiology, Chinese People's Liberation Army General Hospital, Beijing, China; ^3^Department of Ultrasound, Hainan Hospital of Chinese People's Liberation Army General Hospital, Sanya, China; ^4^Central Laboratory, Hainan Hospital of Chinese People's Liberation Army General Hospital, Sanya, China; ^5^Department of Health Medicine, Hainan Hospital of Chinese People's Liberation Army General Hospital, Sanya, China; ^6^Department of Epidemiology, School of Public Health, Southern Medical University, Guangzhou, China; ^7^Center for the Study of Aging and Human Development and Geriatrics Division, Medical School of Duke University, Durham, NC, United States; ^8^Center for Healthy Aging and Development Studies, National School of Development, Peking University, Beijing, China

**Keywords:** ataxia-telangiectasia mutated gene, human lifespan, mutant rs189037, ventricular thickness, human longevity

## Abstract

In the current study, we aimed to determine the association of single nucleotide polymorphism rs189037 in ataxia-telangiectasia mutated (*ATM*) gene with cardiac structure and human longevity. Based on the China Hainan Centenarian Cohort Study performed in 18 cities and counties of Hainan Province, China, the current study enrolled 547 centenarians, 250 young participants aged 20–45 years, and 250 middle-aged and elderly participants aged 46–90 years. The frequency of TT genotype was significantly higher and that of CC genotype was significantly lower in middle-aged and elderly participants than in young (*P* = 0.012) and centenarian (*P* = 0.041) participants. There were no significant differences in the genotype and allele frequencies of SNP rs189037 between young and centenarian participants. Compared with CT genotype, TT genotype was positively and significantly associated with interventricular septum thickness (IVST) and left ventricular posterior wall thickness (LVPWT) in centenarian (IVST: *P* = 0.049; LVPWT: *P* = 0.047) and middle-aged and elderly (IVST: *P* = 0.008; LVPWT: *P* = 0.004) participants. Compared with CC genotype, TT genotype was positively and significantly associated with LVPWT in centenarian (*P* = 0.030) and middle-aged and elderly (*P* = 0.013) participants. Compared with CC genotype, CT genotype was negatively and significantly associated with left ventricular end-diastolic diameter (LVEDD) in centenarian (*P* = 0.011) and middle-aged and elderly (*P* = 0.040) participants. The current study demonstrated that mutant rs189037 in the *ATM* gene was more commonly identified in middle-aged and elderly participants than in young and centenarian participants, was significantly associated with increased left ventricular wall thickness and volume, and could induce left ventricular eccentric hypertrophy and shorten human lifespan. Therefore, rs189037 without mutation might be an indicator of youth health and successful aging, whereas mutant rs189037 might hinder human longevity.

## Introduction

The average lifespan of a human ranges from ~65 to 85 years, and the frequency of centenarians is 1 in 10,000 worldwide (1). In studies conducted on twins, human longevity has been indicated to be moderately heritable, and different genetic mutations account for ~25% of human longevity (2). Although studies have been performed to evaluate the role of genetic mutations in human longevity, only the apolipoprotein E (*APOE*) gene has been analyzed in several populations (3, 4). Following the discovery of the association between *APOE* gene and human longevity, lipid metabolism has become the most studied pathway; however, there is some evidence that human longevity is associated with an increased resistance to oxidative stress (5). Chen and colleagues have reported the relationship of centenarian longevity with single nucleotide polymorphism (SNP) rs189037 of the ataxia-telangiectasia mutated (*ATM*) gene (6). The protein encoded by *ATM* gene is resistant to oxidative stress owing to its role in the detection of reactive oxygen species (ROS)-induced lesions and repair of DNA defects (7, 8). ROS is now considered to contribute to senescence primarily by inducing DNA double-strand breaks (DSBs) and causing telomere dysfunction (9). *ATM* gene detects DNA damage and phosphorylates and activates downstream kinases, such as checkpoint kinases ChK1 and ChK2 and P53 (10). Moreover, an antioxidant system exists, which can reduce ROS levels. *ATM* gene plays a crucial role in balancing redox state, and it is widely expressed in many tissues, including the brain, skin, and endothelial cells (11). *ATM* expression and function are markedly decreased in aged mice. Patients with mutations in *ATM* gene present with insulin resistance, growth retardation, immune deficiency, and increased susceptibility to cancer. These observations suggest that *ATM* is critical to human lifespan (12). Studies have investigated the association between mutations in *ATM* gene and exceptional human longevity, but to the best of our knowledge, no studies have been conducted to assess the influence of the gene across human lifespan (6). The genetic basis for influencing human lifespan could improve the understanding of aging mechanisms, thus providing new insights and targets to avoid structural abnormality and promote successful aging. An abnormal cardiac structure is responsible for increased mortality rates and a significant obstacle in improving human longevity. At present, studies on SNPs in *ATM* gene have primarily focused on cancer and lifespan, and limited information regarding the specific effects of mutant rs189037 in *ATM* gene on cardiac structure and human longevity is available. In the current study, we aimed to determine the association of SNP rs189037 in *ATM* gene with cardiac structure and human longevity.

## Materials and Methods

### Population

As a population-based study, the China Hainan Centenarian Cohort Study (CHCCS) was performed in 18 cities and counties of Hainan Province, China, from July 2014 to December 2016 (13). It enrolled 547 centenarians identified by the National Civil Registry from the Hainan Civil Affairs Bureau. Age was ascertained from national identification cards. Among 547 centenarians, the median age was 102 years (101, 104; range: 100–116 years). The current study enrolled 250 young participants aged 20–45 years [median age: 32 years (28, 37) years; range: 22–45 years] and 250 middle-aged and elderly participants aged 46–90 years [median age: 64 years (55, 73); range: 46–89 years] in the same geographical area. These groups were carefully interviewed to exclude the presence of relatives with exceptional longevity in the past generations. The current study was approved by the Ethics Committee of Hainan Hospital of Chinese People's Liberation Army General Hospital (Sanya, Hainan; Number: 301hn11201601) and conducted in accordance with the provisions of the Helsinki Declaration. Written informed consents were obtained from all participants prior to the start of this study.

### Procedures

Based on a standardized protocol, in-person interviews, physical examinations, and blood analyses were conducted by a well-trained research team of the Chinese People's Liberation Army General Hospital by home visits. This interdisciplinary research team included internists, geriatricians, cardiologists, endocrinologists, nephrologists, and nurses. Blood pressure was measured on the right arm of participants using a calibrated desktop sphygmomanometer (Yuwell Medical Equipment & Supply Co., Ltd., Jiangsu, China). Participants sat on a chair for 5 min with feet on the floor and arm supported at heart level. Systolic blood pressure (SBP) and diastolic blood pressure (DBP) were recorded at the first and fifth Korotkoff sounds, respectively. Participants with SBP ≥140 mmHg or DBP ≥90 mmHg or those receiving medications for treatment of hypertension were defined as patients with hypertension (14). Ultrasound (Philips CX50, Philips Medical Systems, Andover, MA, USA) was used to measure the parameters of cardiac structure and function by experienced radiologists who were unaware of clinical and laboratory data (15). All participants were asked to lie down in a supine position, and chest was scanned using a 1–5-MHz cardiac probe (S5–1).

Samples of venous blood were routinely collected by venipuncture and stored at 4°C and delivered to the central laboratory at the Department of Biochemistry, Hainan Hospital of Chinese People's Liberation Army General Hospital, within 4 h. Serum levels of triglyceride (TG), total cholesterol (TC), high-density lipoprotein cholesterol (HDL-C), and fasting blood glucose (FBG) were measured by enzymatic assays (Roche Products Ltd, Basel, Switzerland) using a fully automatic biochemical autoanalyzer (Cobas c702; Roche Products Ltd, Basel, Switzerland), and low-density lipoprotein cholesterol (LDL-C) level was calculated. Qualified technicians who performed laboratory analyses were blinded to clinical data.

### Genotyping

**Figure 1:**
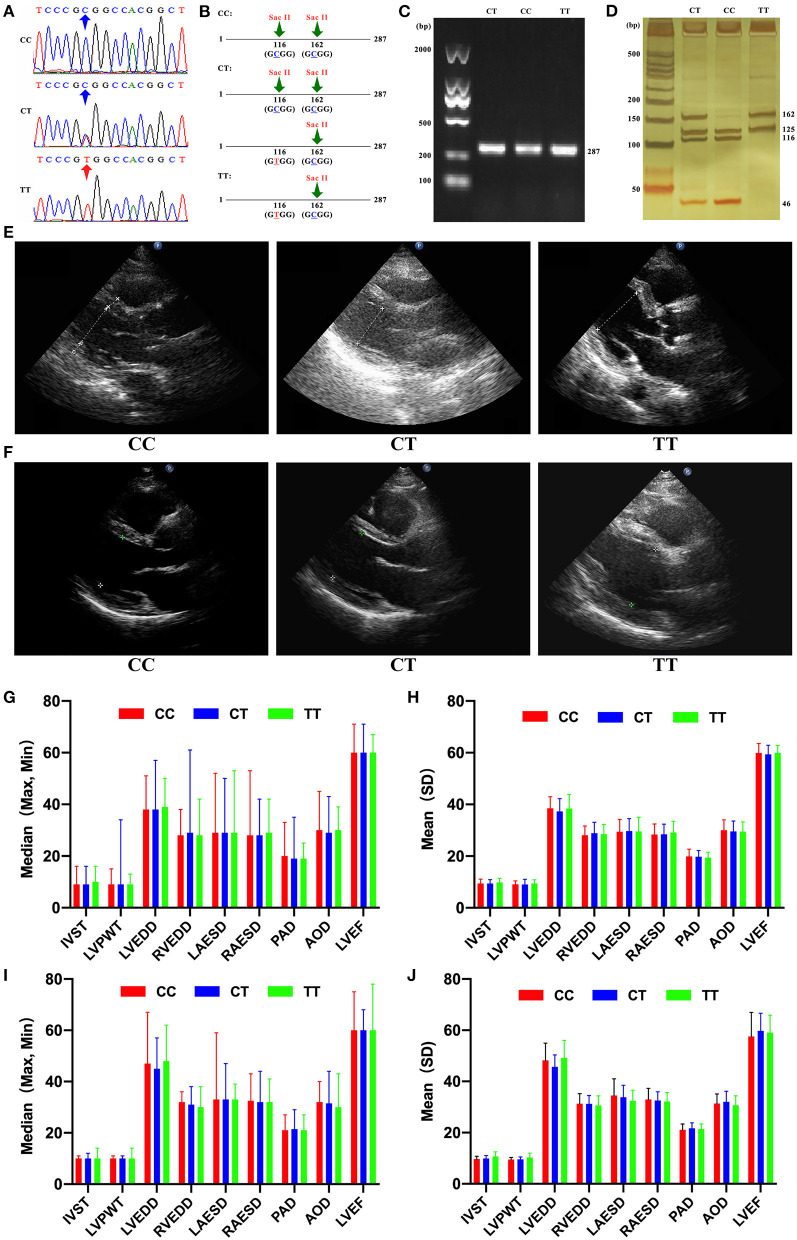
**(A)** DNA sequencing of ataxia-telangiectasia-mutated (*ATM*) gene with different genotypes of single nucleotide polymorphism (SNP) rs189037. **(B)** *ATM* promoter region contains two SacII restriction enzyme sites, and one of them is present in SNP rs189037 (C/T) only when T → C institution at nucleotide 116 occurs. **(C)** Amplification products (287 bp) of *ATM* gene stained with gelgreen on a 1% agarose gel. **(D)** Polymerase chain reaction products digested with SacII and stained with silver nitrate on a 20% polyacrylamide gel. **(E)** Cardiac structure shown by ultrasound in centenarians with different genotypes of SNP rs189037. **(F)** Cardiac structure shown by ultrasound in middle-aged and elderly participants with different genotypes of SNP rs189037. **(G)** Median [maximum (Max), minimum (Min)] of cardiac structure and function in centenarians with different genotypes of SNP rs189037. **(H)** Mean [standard deviation (SD)] of cardiac structure and function in centenarians with different genotypes of SNP rs189037. **(I)** Median [maximum (Max), minimum (Min)] of cardiac structure and function in middle-aged and elderly participants with different genotypes of SNP rs189037. **(J)** Mean [standard deviation (SD)] of cardiac structure and function in middle-aged and elderly participants with different genotypes of SNP rs189037.

Genomic DNA was isolated from peripheral blood samples using a QIAamp DNA Mini kit and eluted using 60 μl of buffer AE (Qiagen, Hilden, Germany). The concentration and purity of DNA were assessed using the Gen5 CHS 2.01 Software on the Eon microplate spectrophotometer (BioTeK, Winooski, Vermont, USA). SNP rs189037 in *ATM* gene was genotyped in all participants using polymerase chain reaction-restriction fragment length polymorphism (PCR-RFLP) and confirmed in 180 participants (>15%) by sequencing using a 3730XL automatic DNA sequencer (Applied Biosystems, Thermo Fisher Scientific, Waltham, MA, USA). The rate of concurrence of both methods was 100%, indicating PCR-RFLP as a reliable method. Primer design and DNA sequencing were performed by BGI (Shenzhen, China; Figure 1A). The forward and reverse primers were 5′ -GCTGCTTGGCGTTGCTTC-3′and 5′-CATGAGATTGGCGGTCTGG3′ (Invitrogen, Thermo Fisher Scientific, Waltham, MA, USA), respectively. PCR was performed in 12.5 μl total volume containing 1 μl of genomic DNA. The following PCR procedures were performed: an initial denaturation step at 94°C for 4 min, followed by 35 cycles of denaturation at 94°C for 30 s, annealing at 56°C for 30 s, elongation for 30 s at 72°C, and a final extension at 72°C for 5 min. *ATM* promoter region contains two Sac II restriction enzyme sites, and one of them is present in SNP rs189037 (C/T) only when T → C substitution at nucleotide 116 occurs (Figure 1B). Amplification products (287 bp) of *ATM* gene were visualized on a 1% agarose gel by staining with gelgreen (Tiangen, Beijing, China; Figure 1C). PCR products were digested with SacII (New England Biolabs, Ipswich, MA, USA) at 37°C overnight and stained with silver nitrate after resolution by electrophoresis on a 20% polyacrylamide gel (Figure 1D). All genotypes were determined as follows: three fragments of 46, 116, and 125 bp, respectively, for CC genotype; two fragments of 125 and 162 bp, respectively, for TT genotype; and four fragments of 46, 116, 125, and 162 bp, respectively, for CT genotype.

### Statistical Analyses

Statistical analyses were performed using SPSS software (version 17.0, SPSS Inc., Chicago, IL, USA). Data are presented as means and standard deviations (continuous variables with normal distributions), medians and interquartile ranges (continuous variables with skewed distributions), and numbers and percentages (categorical variables). Continuous data were compared using analysis of variance (ANOVA; for variables with normal distribution) or Kruskal-Wallis test (for variables with skewed distribution). Categorical data, including genotype and allele frequencies, were compared using Chi-square test. Hardy-Weinberg equilibrium was tested by Chi-square test. Multivariate logistic regression was applied to analyze the associations between the genotypes of SNP rs189037 and age groups with adjustments for sex; nationality; hypertension; and FBG, TC, TG, HDL-C, and LDL-C levels. Multivariate logistic regression was applied to analyze the associations between the genotypes of SNP rs189037 and cardiac structure and function with adjustments for age; sex; nationality; hypertension; and FBG, TC, TG, HDL-C, and LDL-C levels. Statistical significance was set at a two-sided *P* < 0.05.

## Results

Table 1 describes the basic characteristics and genotype distribution of SNP rs189037 in young, middle-aged and elderly, and centenarian participants. The frequency of TT genotype was significantly higher and that of CC genotype was significantly lower in middle-aged and elderly participants than in young (*P* = 0.012) and centenarian (*P* = 0.041) participants. C allele in middle-aged and elderly participants was significantly less frequent compared with young (*P* = 0.005) and centenarian (*P* = 0.019) participants. There were no significant differences in the genotype and allele frequencies of SNP rs189037 between young and centenarian participants. Distributions of genotype frequencies in young (*P* = 0.232), middle-aged and elderly (*P* = 0.471), and centenarian (*P* = 0.982) participants were compatible with the Hardy-Weinberg equilibrium (*P* > 0.05).

**Table 1:** Basic characteristics of participants with different genotypes of rs189037.

**Characteristics**	**Young**	**Middle-aged and elderly**	**Centenarians**	***P*^**a**^**	***P*^**b**^**	***P*^**c**^**	***P*^**d**^**
	**(*n* = 250)**	**(*n* = 250)**	**(*n* = 547)**				
Age (year)^e^	32 (28, 37)	64 (55, 73)	102 (101, 104)	<0.001	<0.001	<0.001	<0.001
Males (%)	137 (54.8)	156 (62.4)	93 (17.0)	<0.001	0.085	<0.001	<0.001
Han nationality (%)	198 (79.2)	185 (74.0)	471 (86.1)	<0.001	0.170	0.014	<0.001
Hypertension (%)	3 (1.2)	124 (49.6)	462 (84.5)	<0.001	<0.001	<0.001	<0.001
Systolic blood pressure (mmHg)^e^	109 (102, 116)	123 (113, 136)	151 (135, 171)	<0.001	<0.001	<0.001	<0.001
Diastolic blood pressure (mmHg)^e^	71 (66, 77)	69 (62, 76)	75 (66, 83)	<0.001	0.009	<0.001	<0.001
Fasting blood glucose (mmol/L)^e^	4.68 (4.44, 4.92)	5.93 (5.16, 8.07)	4.82 (4.20, 5.74)	<0.001	<0.001	0.015	<0.001
Triglyceride (mmol/L)^e^	1.03 (0.75, 1.57)	1.37 (1.03, 1.94)	1.03 (0.80, 1.43)	<0.001	<0.001	0.946	<0.001
Total cholesterol (mmol/L)^e^	4.70 (4.05, 5.28)	4.07 (3.25, 4.87)	4.57 (4.06, 5.18)	<0.001	<0.001	0.482	<0.001
Low-density lipoprotein cholesterol (mmol/L)^e^	2.76 (2.29, 3.23)	2.41 (1.81, 3.16)	2.71 (2.29, 3.24)	<0.001	<0.001	0.601	<0.001
High-density lipoprotein cholesterol (mmol/L)^e^	1.40 (1.17, 1.87)	1.08 (0.85, 1.41)	1.40 (1.18, 1.67)	<0.001	<0.001	0.082	<0.001
C (%)	312 (62.4)	268 (53.6)	655 (59.9)	0.013	0.005	0.019	0.338
CC [*n* (%)]	91 (36.4)	67 (26.8)	195 (35.6)	0.029	0.012	0.041	0.266
CT [*n* (%)]	130 (52.0)	134 (53.6)	265 (48.4)				
TT [*n* (%)]	29 (11.6)	49 (19.6)	87 (15.9)				
Hardy–Weinberg equilibrium	0.232	0.471	0.982				

a *Centenarians/middle-aged and elderly/young*.

b *Middle-aged and elderly/young*.

c *Centenarians/young*.

d *Centenarians/middle-aged and elderly*.

e *Median (interquartile range)*.

Table 2 shows the multiple associations of age groups with different genotypes of SNP rs189037. Compared with CC genotype, TT genotype was positively and significantly associated with middle-aged and elderly/young participants [Exp(B) (95% CI): 3.303 (1.107–9.859); *P* = 0.032]. The genotypes of SNP rs189037 were not significantly associated with centenarian/young participants.

**Table 2:** Associations of age group with different genotypes of rs189037.

**Characteristics**	**TT/CT**		**TT/CC**		**TT/CT+CC**		**CT/CC**		**CT/TT+CC**	
**Multivariate logistic regression^**a**^**	**Exp(B) (95% CI)**	***P***	**Exp(B) (95% CI)**	***P***	**Exp(B) (95% CI)**	***P***	**Exp(B) (95% CI)**	***P***	**Exp(B) (95% CI)**	***P***
Middle-aged and elderly/young	1.317 (0.496–3.493)	0.580	3.303 (1.107–9.859)	0.032	1.781 (0.811–3.908)	0.150	1.886 (0.929–3.829)	0.079	1.355 (0.747–2.460)	0.317
Centenarians/young	0.609 (0.232–1.599)	0.313	0.947 (0.340–2.638)	0.918	0.800 (0.329–1.946)	0.623	1.301 (0.741–2.284)	0.359	1.327 (0.781–2.257)	0.296
Centenarians/middle-aged and elderly	0.834 (0.435–1.601)	0.586	0.456 (0.206–1.009)	0.053	0.694 (0.372–1.295)	0.251	0.605 (0.351–1.040)	0.069	0.768 (0.476–1.239)	0.279

a *With adjustment of sex, nationality, hypertension, fasting blood glucose, total cholesterol, triglyceride, high-density lipoprotein cholesterol, and low-density lipoprotein cholesterol*.

The basic characteristics of centenarians with different genotypes of SNP rs189037 are presented in Table 3. Centenarians with TT and CT genotypes showed significant differences in left ventricular posterior wall thickness (LVPWT; *P* = 0.004). Centenarians with TT and CC genotypes showed significant differences in LVPWT (*P* = 0.047; Figures 1E,G,H). Centenarians with CT and CC genotypes showed significant differences in LVEDD (*P* = 0.008).

**Table 3:** Basic characteristics of centenarians with different genotypes of rs189037.

**Characteristics**	**TT**	**CT**	**C**	***P*^**a**^**	***P*^**b**^**	***P*^**c**^**	***P*^**d**^**
	**(*n* = 87)**	**(*n* = 265)**	**(*n* = 195)**				
Age (year)^e^	102 (101, 104)	102 (101, 104)	102 (101, 104)	0.319	0.203	0.806	0.172
Males (%)	9 (10.3)	51 (19.2)	33 (16.9)	0.159	0.055	0.152	0.524
Han nationality (%)	80 (92.0)	223 (84.2)	168 (86.2)	0.189	0.068	0.167	0.552
Hypertension (%)	80 (92.0)	214 (80.8)	168 (86.2)	0.031	0.015	0.167	0.127
Systolic blood pressure (mmHg)^e^	150 (137, 169)	150 (132, 170)	151 (136, 174)	0.647	0.553	0.948	0.382
Diastolic blood pressure (mmHg)^e^	74 (68, 85)	76 (66, 82)	75 (65, 83)	0.970	0.798	0.863	0.953
Fasting blood glucose (mmol/L)^e^	4.64 (4.22, 5.75)	4.91 (4.19, 5.72)	4.88 (4.20, 5.76)	0.612	0.395	0.332	0.846
Triglyceride (mmol/L)^e^	1.05 (0.80, 1.36)	1.04 (0.81, 1.47)	1.01 (0.78, 1.43)	0.770	0.971	0.585	0.508
Total cholesterol (mmol/L)^e^	4.67 (4.16, 5.49)	4.56 (4.00, 5.15)	4.54 (4.07, 5.21)	0.346	0.183	0.171	0.908
Low-density lipoprotein cholesterol (mmol/L)^e^	2.88 (2.26, 3.47)	2.71 (2.30, 3.23)	2.66 (2.28, 3.16)	0.354	0.311	0.150	0.524
High-density lipoprotein cholesterol (mmol/L)^e^	1.41 (1.16, 1.65)	1.39 (1.19, 1.66)	1.40 (1.17, 1.69)	0.871	0.840	0.622	0.702
Interventricular septum thickness (mm)^e^	10 (9, 11)	9 (8, 10)	9 (8, 10)	0.142	0.068	0.069	0.802
Left ventricular posterior wall thickness (mm)^e^	9 (8, 10)	9 (8, 10)	9 (8, 10)	0.016	0.004	0.047	0.227
Left ventricular end-diastolic diameter (mm)^e^	39 (34, 42)	38 (34, 40)	38 (36, 41)	0.016	0.052	0.875	0.008
Right ventricle end-diastolic diameter (mm)^e^	28 (26, 31)	29 (26, 31)	28 (26, 31)	0.235	0.540	0.551	0.086
Left atrium end-systolic diameter (mm)^e^	29 (26, 32)	29 (27, 32)	29 (26, 31)	0.663	0.616	0.883	0.380
Right atrium end-systolic diameter (mm)^e^	29 (27, 32)	28 (26, 31)	28 (26, 31)	0.383	0.257	0.179	0.658
Pulmonary artery diameter (mm)^e^	19 (18, 21)	19 (18, 21)	20 (18, 21)	0.288	0.298	0.121	0.417
Aorta diameter (mm)^e^	30 (27, 32)	29 (27, 32)	30 (27, 33)	0.493	0.922	0.437	0.253
Left ventricular ejection fraction (%)^e^	60 (60, 60)	60 (58, 60)	60 (60, 60)	0.186	0.149	0.860	0.125

a *TT/CT/CC*.

b *TT/CT*.

c *TT/CC*.

d *CT/CC*.

e *Median (interquartile range)*.

Table 4 shows multiple associations of cardiac structure and function with different genotypes of SNP rs189037 in centenarians. Compared with CT genotype, TT genotype was positively and significantly associated with IVS [Exp(B) (95% CI): 1.181 (1.001–1.394); *P* = 0.049] and LVPWT [Exp(B) (95% CI): 1.152 (1.002–1.324); *P* = 0.047] in centenarians. Compared with CC genotype, TT genotype was positively and significantly associated with LVPWT [Exp(B) (95% CI): 1.244 (1.021–1.517); *P* = 0.030] in centenarians. Compared with CC genotype, CT genotype was negatively and significantly associated with LVEDD [Exp(B) (95% CI): 0.947 (0.908–0.988); *P* = 0.011] and positively and significantly associated with RVEDD [Exp(B) (95% CI): 1.064 (1.009–1.123); *P* = 0.022] in centenarians.

**Table 4:** Associations of cardiac structure and function with different genotypes of rs189037 in the centenarians.

**Characteristics**	**TT/CT**		**TT/CC**		**TT/CT+CC**		**CT/CC**		**CT/TT+CC**	
**Multivariate logistic regression^**a**^**	**Exp(B) (95% CI)**	***P***	**Exp(B) (95% CI)**	***P***	**Exp(B) (95% CI)**	***P***	**Exp(B) (95% CI)**	***P***	**Exp(B) (95% CI)**	***P***
Interventricular septum thickness (mm)	1.181 (1.001–1.394)	0.049	1.158 (0.987–1.358)	0.071	1.154 (0.999–1.333)	0.051	1.002 (0.888–1.131)	0.971	0.953 (0.854–1.064)	0.394
Left ventricular posterior wall thickness (mm)	1.152 (1.002–1.324)	0.047	1.244 (1.021–1.517)	0.030	1.140 (1.009–1.288)	0.036	0.978 (0.879–1.089)	0.686	0.929 (0.832–1.037)	0.190
Left ventricular end-diastolic diameter (mm)	1.055 (0.999–1.113)	0.055	1.007 (0.952–1.064)	0.818	1.031 (0.980–1.084)	0.235	0.947 (0.908–0.988)	0.011	0.948 (0.914–0.985)	0.006
Right ventricle end-diastolic diameter (mm)	0.988 (0.923–1.057)	0.716	1.059 (0.984–1.141)	0.127	1.015 (0.954–1.080)	0.644	1.064 (1.009–1.123)	0.022	1.047 (0.999–1.097)	0.057
Left atrium end-systolic diameter (mm)	0.994 (0.946–1.045)	0.822	1.002 (0.951–1.054)	0.951	0.997 (0.951–1.045)	0.895	1.016 (0.976–1.058)	0.434	1.013 (0.978–1.049)	0.464
Right atrium end-systolic diameter (mm)	1.048 (0.984–1.116)	0.143	1.060 (0.994–1.130)	0.074	1.053 (0.995–1.115)	0.074	1.009 (0.961–1.060)	0.707	0.991 (0.949–1.035)	0.687
Pulmonary artery diameter (mm)	0.939 (0.837–1.053)	0.280	0.926 (0.830–1.033)	0.168	0.932 (0.841–1.033)	0.177	0.977 (0.907–1.052)	0.532	0.999 (0.933–1.070)	0.978
Aorta diameter (mm)	0.999 (0.936–1.065)	0.964	0.970 (0.907–1.038)	0.379	0.985 (0.929–1.046)	0.630	0.972 (0.927–1.019)	0.238	0.980 (0.939–1.024)	0.370
Left ventricular ejection fraction (%)	1.035 (0.960–1.116)	0.368	0.985 (0.913–1.064)	0.705	1.011 (0.945–1.082)	0.746	0.955 (0.905–1.008)	0.095	0.958 (0.911–1.007)	0.094

a *With adjustment of age, sex, nationality, hypertension, fasting blood glucose, total cholesterol, triglyceride, high-density lipoprotein cholesterol, and low-density lipoprotein cholesterol*.

The basic characteristics of middle-aged and elderly participants with different genotypes of SNP rs189037 are presented in Table 5. Middle-aged and elderly participants with TT and CT genotypes showed significant differences in LVPWT (*P* = 0.037), LVEDD (*P* = 0.015), and left ventricular ejection fraction (LVEF, *P* = 0.032). Middle-aged and elderly participants with TT and CC genotypes showed significant differences in LVPWT (*P* = 0.043; Figures 1F,I,J).

**Table 5:** Basic characteristics of middle-aged and elderly with different genotypes of rs189037.

**Characteristics**	**TT**	**CT**	**CC**	***P*^a^**	***P*^b^**	***P*^c^**	***P*^d^**
	**(*n* = 87)**	**(*n* = 265)**	**(*n* = 195)**				
Age (year)^e^	65 (58, 73)	64 (56, 73)	63 (53, 73)	0.888	0.758	0.544	0.905
Males (%)	30 (34.5)	82 (30.9)	44 (22.6)	0.059	0.539	0.036	0.046
Han nationality (%)	37 (42.5)	101 (38.1)	47 (24.1)	0.001	0.464	0.001	0.002
Hypertension (%)	24 (27.6)	66 (24.9)	34 (17.4)	0.082	0.619	0.051	0.055
Systolic blood pressure (mmHg)^e^	129 (117, 139)	122 (109, 131)	128 (114, 142)	0.054	0.053	0.997	0.051
Diastolic blood pressure (mmHg)^e^	67 (60, 76)	68 (62, 76)	70 (61, 79)	0.617	0.369	0.409	0.794
Fasting blood glucose (mmol/L)^e^	5.88 (5.24, 8.29)	5.80 (5.04, 7.99)	6.33 (5.26, 8.23)	0.572	0.474	0.811	0.345
Triglyceride (mmol/L)^e^	1.29 (1.04, 1.66)	1.33 (0.94, 1.94)	1.58 (1.14, 2.08)	0.172	0.644	0.058	0.143
Total cholesterol (mmol/L)^e^	3.61 (3.02, 4.44)	4.00 (3.25, 4.89)	4.43 (3.45, 5.17)	0.044	0.125	0.015	0.141
Low-density lipoprotein cholesterol (mmol/L)^e^	2.21 (1.65, 2.77)	2.41 (1.80, 3.11)	2.74 (1.98, 3.37)	0.037	0.251	0.013	0.066
High-density lipoprotein cholesterol (mmol/L)^e^	0.99 (0.81, 1.34)	1.10 (0.86, 1.44)	1.10 (0.86, 1.48)	0.397	0.247	0.186	0.796
Interventricular septum thickness (mm)^e^	10 (9, 13)	10 (9, 11)	10 (9, 10)	0.120	0.090	0.062	0.435
Left ventricular posterior wall thickness (mm)^e^	10 (9, 12)	10 (9, 10)	10 (9, 10)	0.067	0.037	0.043	0.637
Left ventricular end-diastolic diameter (mm)^e^	48 (44, 56)	45 (43, 48)	47 (44, 51)	0.034	0.015	0.485	0.100
Right ventricle end-diastolic diameter (mm)^e^	30 (28, 34)	31 (28, 34)	32 (30, 34)	0.537	0.515	0.264	0.511
Left atrium end-systolic diameter (mm)^e^	33 (31, 35)	33 (31, 36)	33 (31, 36)	0.854	0.628	0.599	0.906
Right atrium end-systolic diameter (mm)^e^	32 (30, 34)	32 (30, 35)	33 (31, 36)	0.553	0.601	0.283	0.456
Pulmonary artery diameter (mm)^e^	21 (20, 23)	22 (20, 23)	21 (20, 23)	0.425	0.654	0.468	0.198
Aorta diameter (mm)^e^	30 (29, 32)	32 (29, 34)	32 (29, 34)	0.291	0.116	0.373	0.568
Left ventricular ejection fraction (%)^e^	60 (56, 60)	60 (60, 65)	60 (55, 63)	0.070	0.032	0.697	0.125

a *TT/CT/CC*.

b *TT/CT*.

c *TT/CC*.

d *CT/CC*.

e *Median (interquartile range)*.

Table 6 shows multiple associations of cardiac structure and function with different genotypes of SNP rs189037 in middle-aged and elderly participants. Compared with CT genotype, TT genotype was positively and significantly associated with IVST [Exp(B) (95% CI): 1.739 (1.156–2.615); *P* = 0.008], LVPWT [Exp(B) (95% CI): 2.016 (1.244–3.267); *P* = 0.004], and LVEDD [Exp(B) (95% CI): 1.207 (1.070–1.361); *P* = 0.002] in middle-aged and elderly participants. Compared with CC genotype, TT genotype was positively and significantly associated with IVST [Exp(B) (95% CI): 1.801 (1.141–2.843); *P* = 0.012] and LVPWT [Exp(B) (95% CI): 1.925 (1.145–3.236); *P* = 0.013] in middle-aged and elderly participants. Compared with CC genotype, CT genotype was negatively and significantly associated with LVEDD [Exp(B) (95% CI): 0.909 (0.829–0.996); *P* = 0.040] in middle-aged and elderly participants.

**Table 6:** Associations of cardiac structure and function with different genotypes of rs189037 in the middle-aged and elderly.

**Characteristics**	**TT/CT**		**TT/CC**		**TT/CT+CC**		**CT/CC**		**CT/TT+CC**	
**Multivariate logistic regression^a^**	**Exp(B) (95% CI)**	***P***	**Exp(B) (95% CI)**	***P***	**Exp(B) (95% CI)**	***P***	**Exp(B) (95% CI)**	***P***	**Exp(B) (95% CI)**	***P***
Interventricular septum thickness (mm)	1.739 (1.156–2.615)	0.008	1.801 (1.141–2.843)	0.012	1.876 (1.260–2.792)	0.002	1.310 (0.876–1.960)	0.188	0.886 (0.671–1.170)	0.393
Left ventricular posterior wall thickness (mm)	2.016 (1.244–3.267)	0.004	1.925 (1.145–3.236)	0.013	2.070 (1.318–3.250)	0.002	1.161 (0.698–1.931)	0.565	0.776 (0.549–1.097)	0.151
Left ventricular end-diastolic diameter (mm)	1.207 (1.070–1.361)	0.002	1.053 (0.954–1.162)	0.301	1.118 (1.022–1.222)	0.015	0.909 (0.829–0.996)	0.040	0.888 (0.821–0.961)	0.003
Right ventricle end-diastolic diameter (mm)	0.938 (0.806–1.091)	0.405	0.923 (0.766–1.112)	0.399	0.927 (0.800–1.073)	0.310	0.967 (0.844–1.108)	0.630	1.006 (0.900–1.125)	0.915
Left atrium end-systolic diameter (mm)	0.941 (0.823–1.076)	0.373	0.899 (0.792–1.021)	0.100	0.915 (0.817–1.025)	0.125	0.975 (0.897–1.059)	0.545	1.008 (0.936–1.086)	0.832
Right atrium end-systolic diameter (mm)	0.913 (0.772–1.079)	0.287	0.939 (0.780–1.129)	0.502	0.927 (0.801–1.073)	0.309	0.965 (0.855–1.090)	0.570	1.000 (0.899–1.112)	0.996
Pulmonary artery diameter (mm)	0.908 (0.702–1.175)	0.464	1.104 (0.805–1.513)	0.539	0.965 (0.775–1.202)	0.753	1.153 (0.930–1.430)	0.194	1.129 (0.938–1.359)	0.198
Aorta diameter (mm)	0.892 (0.772–1.030)	0.121	0.941 (0.791–1.118)	0.488	0.907 (0.796–1.035)	0.147	1.063 (0.943–1.198)	0.318	1.085 (0.980–1.201)	0.116
Left ventricular ejection fraction (%)	0.958 (0.886–1.036)	0.284	1.044 (0.960–1.134)	0.314	0.992 (0.928–1.060)	0.812	1.050 (0.987–1.117)	0.124	1.046 (0.989–1.105)	0.114

a *With adjustment of age, sex, nationality, hypertension, fasting blood glucose, total cholesterol, triglyceride, high-density lipoprotein cholesterol, and low-density lipoprotein cholesterol*.

## Discussion

In middle-aged and elderly and centenarian participants, the current study demonstrated that CT genotype of SNP rs189037 was significantly associated with reduced LVEDD, while TT genotype was significantly associated with increased IVST, LVPWT, and LVEDD. For SNP rs189037 in *ATM* gene, heterozygous mutation might result in LV concentric development, whereas homozygous mutation might further induce LV eccentric hypertrophy. Moreover, the frequency of TT genotype was similar between young and centenarian participants; however, middle-aged and elderly participants showed significantly higher frequency of TT genotype. SNP rs189037 without mutation might be an indicator of youth health and successful aging, whereas mutant rs189037 might hinder successful aging and human longevity.

Human lifespan is the outcome of multiple processes involving genetic factors; thus, several genetic studies have investigated putative associations between genes and longevity, but to the best of our knowledge, no studies have been conducted to assess the influence of the genes across human lifespan. The current study identified SNP rs189037 without mutation as a biomarker of youth health and successful aging by analyzing genotypic change in *ATM* across human lifespan. ROS can lead to DSBs and telomere dysfunction, which are critical causes of DNA lesions (8). Once DNA lesions occur, the protein encoded by *ATM* gene phosphorylates numerous downstream effectors involved in the G1/S, intra-S, and G2/M checkpoint responses and additional factors involved in DNA repair, including tumor suppressor proteins P53, ChK1, and ChK2 (10). Therefore, *ATM* mutations possibly make individuals susceptible to cancer and are harmful for physiological activity. This suggests that *ATM* gene plays a significant role in prolonging human lifespan.

Accumulating evidence has illustrated that SNPs in gene regulate gene expression, and people with different SNPs show distinct phenotypes (16, 17). A previous study has shown the presence of an AP-2a-binding site around SNP rs189037 in *ATM* gene, which is a transcriptional factor involved in both physiological and pathological processes, such as tumorigenesis and morphogenesis (18). It has been verified in human cells that TT genotype of SNP rs189037 increases *ATM* mRNA expression, and AP-2a represses *ATM* expression in humans by binding to CC genotype (6). It is also believed that balance exists in every activity of the physiological process; mutant SNP in gene alters protein phenotypes, disrupts physiological balance, and causes pathological changes. For example, mutant SNP rs189037 in *ATM* gene or consistently high activity of P53 results in abnormal cardiac structure or premature senescence of individuals (19). The current study reported that SNP rs189037 without mutation might prevent the development of an abnormal cardiac structure and death at an early age; hence, it is not only an indicator and biomarker of youth health and successful aging but also promotes successful aging and prolongs human lifespan.

Aging is a complicated process affected by several genetic and non-genetic factors (1). Genes are the predominant factors responsible for aging, and SNPs associated with aging might exist. Mice with deficient *ATM* gene have been shown to experience accelerated aging (20). As a regulator of oxidative stress and sensor of DNA damage, *ATM* gene prevents cellular senescence, death, or cell cycle arrest; in addition, it protects individuals from ROS-induced DNA damage. Ding et al. have described an association of SNP rs189037 in *ATM* gene with the proportion of coronary artery disease in Chinese Han population and demonstrated a low proportion of coronary artery disease in participants harboring TT genotype (21). In the current study, the young and centenarian participants showed significantly lower frequency of mutant rs189037 in *ATM* gene than middle-aged and elderly participants. Individuals without mutant rs189037 in *ATM* gene may age later and live long, and reversing mutant rs189037 might be a suitable strategy to delay human aging and promote human longevity.

## Conclusions

The current study demonstrated that mutant SNP rs189037 in *ATM* gene was more commonly identified in the middle-aged and elderly participants than in young participants and centenarians. It was significantly associated with increased LV thickness and volume and might induce LV eccentric hypertrophy and shorten human lifespan.

## Data Availability Statement

The original contributions presented in the study are included in the article/Supplementary Material, further inquiries can be directed to the corresponding authors.

## Ethics Statement

The studies involving human participants were reviewed and approved by Ethics Committee of the Hainan Hospital of the Chinese People's Liberation Army General Hospital (Sanya, Hainan; No: 301hn11201601). The patients/participants provided their written informed consent to participate in this study.

## Author Contributions

SF, JH, XC, BL, HS, JD, YJZ, YY, and YLZ contributed to the study design, conducted the data collection and analyses, and drafted the paper. All authors contributed to the article and approved the submitted version.

## Conflict of Interest

The authors declare that the research was conducted in the absence of any commercial or financial relationships that could be construed as a potential conflict of interest.

## Abbreviations

ApoE: apolipoprotein E
ATM: ataxia-telangiectasia mutated
ChK: checkpoint kinases
CHCCS: China Hainan Centenarian Cohort Study
DBP: diastolic blood pressure
DNA: deoxyribonucleic acid
DSBs: DNA double-strand breaks
FBG: fasting blood glucose
HDL-C: high-density lipoprotein cholesterol
IVST: interventricular septum thickness
LDL-C: low-density lipoprotein cholesterol
LVEDD: left ventricular end-diastolic diameter
LVPWT: left ventricular posterior wall thickness
PCR-RFLP: polymerase chain reaction-restriction fragment length polymorphism
RVEDD: right ventricle end-diastolic diameter
SBP: systolic blood pressure
SNP: single nucleotide polymorphism
TC: total cholesterol
TG: triglyceride.
